# Symbolic Entropy Analysis and Its Applications

**DOI:** 10.3390/e20080568

**Published:** 2018-07-31

**Authors:** Raúl Alcaraz

**Affiliations:** Research Group in Electronic, Biomedical and Telecommunication Engineering, University of Castilla-La Mancha, 13071 Cuenca, Spain; raul.alcaraz@uclm.es; Tel.: +34-969-179-100 (ext. 4847)

**Keywords:** symbolic data analysis, symbolization approaches, symbolic entropy, Transfer entropy, Permutation entropy, Lempel–Ziv complexity

## Abstract

This editorial explains the scope of the special issue and provides a thematic introduction to the contributed papers.

Symbolic data analysis has received a great deal of attention over the last few years and has been applied to many research areas, including astrophysics and geophysics, biology and medicine, fluid flow, chemistry, mechanical systems, artificial intelligence, communication systems, and, recently, data mining and big data [1,2,3]. A fundamental step in this methodology is the quantization of original data into a corresponding sequence of symbols. The resulting time series is then considered a transformed version of the original data, allowing to highlight its temporal information. Indeed, it has been proven that this symbolization procedure can notably improve signal-to-noise ratios in some noisy time series [4]. Moreover, symbolic data analysis also makes communication and numerical computation more efficient and effective, compared with the processing of continuous-valued time series [5].

However, symbolization of a time series always involves information loss and, hence, this process deserves special attention [4]. Classical approaches to this problem consist of subdividing the data range into a finite number of intervals or in an ordinal manner, for example by considering the up and down behavior of subsequent measured values [1,2]. Nonetheless, for both cases the optimal size of the symbol alphabet that retains the most relevant information from the original time series is still a key aspect under debate [6]. To address this challenge, one of the 17 papers published in this Special Issue has introduced a novel symbolization approach, which automatically generates a set of symbols by considering dependencies between the original samples [7].

In a similar line, Li & Roy [8] have proposed another innovative symbolization algorithm by maximizing mutual information of the selected symbols. More precisely, the method is an unsupervised approach that initially establishes a set of partitioning thresholds and then iteratively adds new boundaries whenever mutual information among the symbols increases. In this way, uncertainty in the constructed symbol alphabet was completely removed and high insensitivity to the presence of zero-mean Gaussian and background noises was reached.

After symbolization, information retained in the transformed data has been traditionally quantified through statistical indices (e.g., frequency and transition probabilities between symbols) and information theoretic measures (e.g., Shannon Entropy, Rényi entropy and Conditional entropy), providing well-known symbolic metrics such as Lempel–Ziv complexity (LZC), Permutation entropy (PerEn), and Transfer Entropy (TrEn), among others [1,9]. Interestingly, these indices and some variants have been analyzed in novel applications in the remaining papers collected in this Special Issue.

Given the scientific and social impact of health research, as well as the burgeoning need for ever better diagnostic and therapeutic tools, it is not surprising that many symbolic indices have been used in biomedical applications. Indeed, García-Martínez et al. [10] have customized PerEn, and modified it to be amplitude-aware, for discerning emotional states of calmness and stress from electroencephalogram (EEG) recordings. Both indices reported a very similar discriminant ability of about 65%, which notably increased to 80% when they were combined with another entropy-based metrics that quantify irregularity of time series, such as quadratic sample entropy. According to the authors, the obtained results suggested that both kinds of entropy-based indices highlight complementary neural dynamics, thus revealing a synchronized behavior between frontal and parietal counterparts from both hemispheres of the brain. This finding about how the brain works under different emotions could be helpful for incorporating affective intelligence in brain–computer interfaces.

In a similar way, Shumbayawonda et al. [11] have applied PerEn to magnetoencephalogram recordings with the aim of determining changes due to age and gender in the fingerprint of background brain activity in a large population of healthy subjects. Although the effects of age were seen for all brain areas, no differences were observed in any region for both genders across all ages. As a consequence, the authors concluded that these interesting observations might be useful to assist in the early diagnosis of neurodegenerative conditions.

In the context of out-of-hospital (OHCA) cardiac arrest, PerEn has also been used to predict defibrillation success [12]. To assess the dynamics characterizing poor heart performance during cardiac arrest, this metric, along with other symbolic, non-linear and linear indices, were applied to five second-length electrocardiogram (ECG) intervals just prior to each electrical shock. Although PerEn was not a successful predictor, conditional entropy reached a diagnostic accuracy very similar to the best harbinger, fuzzy entropy. Hence, the authors suggested that symbolic analysis of ECG dynamics could be a promising tool to optimize OHCA treatment, however further experimentation is still required.

A recently proposed variant of PerEn combined the symbolization procedure of this index with the symbol counting approach of common LZC to provide a novel metric able to work with times series showing fast amplitude changes and an unknown origin. This novel algorithm is called Permutation LZC (PLZC) and has been used by Deniz et al. [13] to report notable differences in mouse EEG recordings for between baseline and recovery from sleep deprivation. In contrast to LZC, PLZC revealed an interesting ability to discern activated brain states associated with wakefulness and REM sleep. In both cases, higher levels of complexity were observed in comparison with non-REM sleep. The authors concluded that PLZC could be useful to assess EEG alterations induced by environmental and pharmacological manipulations.

Another modification of LZC has been proposed by Simons & Abásolo [14]. Distance-based LZC (dLZC) was introduced to quantify changes between pairs of EEG channels, so that the index reports higher values for pairs of EEG signals with few sub-sequences in common than for those with a large percentage of similar patterns. Accordingly, the authors noticed that in most brain regions had lower dLZC values for patients suffering from Alzheimer’s disease than for age-matched control subjects, suggesting a more limited richness of the neural information in the dementia patients.

For jointly dealing with several human gait signals, Yu et al. [15] have proposed a multivariate multi-scale symbolic entropy analysis. More precisely, they computed Shannon entropy for the accumulated symbol histogram obtained from several coarse-grained time series to report notable differences between walking conditions for healthy subjects and neurodegenerative patients. In view of this finding, the authors suggested that the proposed tool might be successfully embedded into wearable devices for long-term monitoring of patients with neurodegenerative disorders.

In the final work introducing a biomedical application, Shannon entropy has been used to quantify changes in statistical properties of ultrasound signals induced by fatty infiltration in the liver [16]. Thus, entropy both from ultrasound radio-frequency and uncompressed envelope signals was computed for different levels of fat in the liver. The obtained results showed that fatty infiltration increased signal uncertainty of backscattered echoes from the liver, but Shannon entropy was still able to identify fatty livers with sensitivity, specificity and accuracy values of about 90%. As a consequence, the authors pointed out that ultrasound entropy imaging has the potential for routine use in examination of fatty liver disease.

In a completely different context, Yao et al. [17] have studied information transfer routes among cross-industry and cross-region electricity consumption data through the well-established TrEn. This metric has proven to be highly efficient and robust for quantifying the dominant direction of information flow among time series from structurally identical and non-identical coupled systems. Thus, the authors observed that target and driven industries tend to contain much more information flow than driving ones in the Guangdong Province and, additionally, they are more influential on determining the degree of order of regional industries.

On the other hand, it is worth noting that symbolic analysis also plays a key role in the context of machine learning and two interesting papers have been included in this Special Issue. Duan and Wang [18] have presented an ensemble classification approach, named *k*-dependence Bayesian forest, which induces a specific attribute order and conditional dependencies among attributes. The algorithm was validated on 40 databases, providing better classification outcomes than other common ensemble classifiers. However, despite this sound performance and that Bayesian classifiers have demonstrated competitive classification accuracy in a variety of real-world applications, they are not completely successful for discriminating between high-confidence labels. To alleviate this issue, Sun et al. [19] have proposed an innovative label-driven learning framework, which incorporates three components: a generalist classifier, a refined classification approach by measuring mutual dependence among attributes and, finally, an expert classifier tailored for each high-confidence label. The experiments conducted on several datasets proved that the proposed algorithm performance was better than other well-established Bayesian network classifiers.

Another interesting application of symbolic analysis has been presented by Bat-Erdene et al. [20]. In this work, an approach has been introduced to detect several packing algorithms. Recently, the proportion of packed malware has rapidly grown due to the use of some packing techniques that conceal malware attacks and, hence, the identification and classification of these algorithms are becoming vital for revealing their real intention. Precisely, with the aim of identifying three methods extensively used in malware development—single-layer packing, re-packing and multi-layer packing—the proposed approach converts entropy values of the executable file into symbolic representations, making use of a well-known symbolic aggregate approximation (SAX) methodology. Considering 2196 programs and 19 packing algorithms, the detector reached values of precision, accuracy, and recall of 97.7%, 97.5% and 96.8%, respectively.

From a stricter mathematical point of view, Zhao et al. [21] have inferred a formula of packing pressure of a factor, as well as presenting its application to conformal repellers. Meanwhile, Li et al. [22] have introduced the set of quasi-regular points in countable symbolic space and, moreover, estimated the sizes of those sets using Billingsley–Hausdorff dimension (defined by Gibbs measures). Furthermore, with the aim of clarifying dynamics of some real-world complex systems that are unexplained by classical theories, including phenomena such as combustion, drug delivery or solid component separation in mixtures, Grigorovici et al. [23] have introduced fractal entropy. This novel index was established through non-differentiable Lie groups compatible with a Hamiltonian-type formalism and applied to some physical systems and biological structures.

In the last paper published in the Special Issue, Mladenovic et al. [24] have presented the use of symbolic processing to reduce the number of calculation operations in iteration-based simulation methodologies, as well as to accelerate their computation. The proposed algorithm was validated on two examples—the computation of non-coherent amplitude shift keying with shadowing, interference, and correlated noise; and the estimation of second-order statistics in wireless channels. According to the authors, the method may be easily extrapolated to many other applications where fast computation in one-step simulation runs is required.
